# Effects of salinity and ascorbic acid on growth, water status and antioxidant system in a perennial halophyte

**DOI:** 10.1093/aobpla/plv004

**Published:** 2015-01-19

**Authors:** Abdul Hameed, Salman Gulzar, Irfan Aziz, Tabassum Hussain, Bilquees Gul, M. Ajmal Khan

**Affiliations:** 1 Institute of Sustainable Halophyte Utilization (ISHU), University of Karachi, Karachi 75270, Pakistan; 2 Centre for Sustainable Development, College of Arts and Sciences, Qatar University, PO Box 2713, Doha, Qatar

**Keywords:** Antioxidant, exogenous ascorbic acid application, halophyte, *Limonium stocksii*, salt tolerance

## Abstract

Salinity causes oxidative stress in plants by enhancing production of reactive oxygen species, so that an efficient antioxidant system, of which ascorbic acid (AsA) is a key component, is an essential requirement of tolerance. However, antioxidant responses of plants to salinity vary considerably among species. *Limonium stocksii* is a sub-tropical halophyte found in the coastal marshes from Gujarat (India) to Karachi (Pakistan) but little information exists on its salt resistance. In order to investigate the role of AsA in tolerance, 2-month-old plants were treated with 0 (control), 300 (moderate) and 600 (high) mM NaCl for 30 days with or without exogenous application of AsA (20 mM) or distilled water. Shoot growth of unsprayed plants at moderate salinity was similar to that of controls while at high salinity growth was inhibited substantially. Sap osmolality, AsA concentrations and activities of AsA-dependant antioxidant enzymes increased with increasing salinity. Water spray resulted in some improvement in growth, indicating that the growth promotion by exogenous treatments could partly be attributed to water. However, exogenous application of AsA on plants grown under saline conditions improved growth and AsA dependent antioxidant enzymes more than the water control treatment. Our data show that AsA-dependent antioxidant enzymes play an important role in salinity tolerance of *L. stocksii*.

## Abstract (precis)

*Limonium stocksii* is a potential commercial cut-flower crop for saline areas using brackish water. We therefore were interested to learn about the mechanism of its salinity tolerance. Plants grew well under lower saline conditions (300 mM NaCl) but higher salinities reduced growth. An increase in leaf osmolality and the management of salinity-induced oxidative stress are the key strategies employed. Exogenous AsA application improved the functioning of the AsA-dependent antioxidant system, leading to better growth.

## Introduction

In plants soil salinity causes both osmotic stress and ionic toxicity, which can be lethal under prolonged exposure (Zhu 2001; Munns and Tester 2008; Yu *et al.* 2012). Halophytes survive salinity by sequestering salts in vacuoles and accumulating organic osmolytes in their cytoplasm (Flowers and Colmer 2008; Hameed and Khan 2011; Nedjimi 2014), thus reducing ion toxicity while maintaining osmo-balance. Halophytes may also employ shoot succulence, salt exclusion from roots, salt excretion through specialized salt glands and sequestering excess salt in old leaves to complete their life cycle under salinity (Flowers and Colmer 2008; Shabala and Mackay 2011). High salinity typically compromises carbon fixation, leading to the over reduction of light-harvesting complexes that cause production of reactive oxygen species (ROS) (Ozgur *et al.* 2013; Hamed *et al.* 2014). These ROS are managed within a narrow functionally important range by using enzymatic and non-enzymatic antioxidants (Jithesh *et al.* 2006; Bose *et al.* 2013). High salt stress, however, can make these systems inadequate causing severe injury that may lead to death (Hameed and Khan 2011).

Ascorbic acid (AsA) plays a key role in salt tolerance of many halophytes (Jithesh *et al.* 2006; Hameed *et al.* 2012; Ozgur *et al.* 2013). It quenches ROS directly as well as through the Asada–Halliwell–Foyer pathway (Noctor and Foyer 1998; Gest *et al.* 2013). It also recycles the lipid-soluble antioxidant α-tocopherol (Lushchak and Semchuk 2012). It may also contribute to maintaining photosynthesis, cell-cycle progression, cell wall expansion, gene expression, synthesis of many hormones, anthocyanin and flavonoids (Smirnoff and Wheeler 2000; Arrigoni and De Tullio 2002; Pignocchi and Foyer 2003; Gest *et al.* 2013). AsA is absorbed readily after exogenous application (Younis *et al.* 2010; Hameed *et al.* 2012) and moves within the plant (Franceschi and Tarlyn 2002; Tedone *et al.* 2004; Herschbach *et al.* 2010). Therefore, foliar application of AsA improves salt tolerance of crop plants in a number of ways (Athar *et al.* 2008; Dolatabadian *et al.* 2008; ElHariri *et al.* 2010; Farahat *et al.* 2013), but little information is available on its role in halophytes (Hameed *et al.* 2012).

*Limonium stocksii*, which is found in the coastal areas of Gujarat (India), Sindh and Balochistan (Pakistan) (Bokhari 1973), is a salt-secreting perennial halophyte in Plumbaginaceae. This species is characterized by beautiful pink-purple flowers and has the potential to become a commercially important cut-flower like many other *Limonium* (aka Sea Lavender or Statice) species (http://www.teleflora.com/about-flowers/statice.asp). Profit margins could be significantly enhanced if *L. stocksii* could be grown using seawater and on saline land. Zia *et al.* (2008) reported that it is a highly salt-tolerant species, but the mechanism of its salt tolerance is not very well understood. We hypothesized that AsA would play a key role in improving salt tolerance; therefore, we investigated: (i) the magnitude of oxidative damage, (ii) levels of enzymatic and non-enzymatic antioxidants and (iii) the role of exogenously applied AsA/water, in response to increasing NaCl concentration.

## Methods

### Seed collection and study site

Seed-bearing inflorescences of *L. stocksii* were collected during July 2009 from Hawks Bay, Karachi, Pakistan (24°52′21.87″N, 66°51′24.58″E, 17 ft. altitude, ∼1.5 km away from the sea front). Seeds were separated from the inflorescence and surface sterilized using 1 % sodium hypochlorite for a minute followed by rinsing with distilled water, air-dried and stored at room temperature.

### Growth conditions

Seeds were sown in plastic pots (12 cm diameter) containing sandy soil and sub-irrigated with half-strength modified Hoagland's solution (Epstein 1972) soon after seedling emergence. Salinity treatments [0 (Control), 300 and 600 mM NaCl] were applied at a rate of 150 mM NaCl per day to minimize osmotic shock. Tap water was used daily to compensate for evaporative loss and the irrigation medium was replaced every third day to avoid salt build up. One week after the final salinity concentrations were reached, plant shoots were sprayed until dripping with AsA (20 mM) or distilled water (each containing 0.1 % Tween-20); these treatments continued twice a week until harvest. Unsprayed plants were controls. Five plants per pot with four pots per treatment were used and were harvested after 30 days of salinity treatment.

### Growth parameters

Shoot fresh weight (FW) was measured soon after harvest while dry weight (DW) was determined after placing plant samples in a forced-draft oven at 60 °C for at least 48 h or until constant weight was achieved.

### Leaf water status

Leaf sap osmolality was determined with the help of a vapour pressure osmometer (VAPRO 5520, Wescor Inc., Logan, UT, USA).

Leaf water content, the difference between FW and DW, was calculated on a DW basis using the following formula:

$$\text{Water}\;\text{content}\;(g\;{\text{H}}_{2}\text{O}\;{\text{g}}^{-1}\;\text{DW)}=\frac{\mathrm{FW}-\text{DW}}{\text{DW}}$$

### Osmoprotectants and antioxidants

Free proline and total soluble sugars (TSS) were quantified in hot-water extracts, which were prepared by boiling finely ground dry plant material in distilled water at 100 °C for 1 h (Khan *et al.* 2000). Proline in hot-water extracts was quantified according to Bates *et al.* (1973). Diluted hot-water extract (1 mL) was added to 1 mL of ninhydrin : glacial acetic acid (1 : 1 v/v) mixture in a test tube, followed by heating at 100 °C for 1 h. After cooling in an ice-bath, proline was estimated from the absorbance (at 520 nm; Beckman DU-530 spectrophotometer, Beckman Coulter Inc., Brea, CA, USA) of the chromophore extracted in toluene using a standard curve with pure proline.

Total soluble sugars were determined using anthrone (Yemm and Willis 1954). Diluted hot-water extract was mixed with anthrone reagent and incubated in a water bath at 100 °C for 30 min. The reaction was stopped in an ice bath and absorbance was noted at 630 nm with Beckman DU-530 spectrophotometer.

Ascorbic acid concentration was estimated by using a slightly modified method of Luwe *et al.* (1993). Freshly harvested plant sample (0.5 g) was ground in liquid nitrogen mortar and then homogenized in ice-cold trichloroacetic acid (TCA, 1 % w/v). The homogenate was then centrifuged at 12 000 × g for 20 min at 4 °C and the supernatant (50 µL) mixed with potassium phosphate buffer (0.95 mL, 100 mM, pH 7.0) and ascorbate oxidase (1 μL of 1 Unit µL^−1^). Oxidation of AsA (*ε* = 14.3 mM^−1^ cm^−1^) was then recoded at 265 nm.

### Antioxidant enzymes

Antioxidant enzymes were extracted as described by Polle *et al.* (1994) with some modifications. Fresh plant samples (0.5 g) were powdered in liquid nitrogen mortar and homogenized in potassium phosphate buffer (5 mL of 100 mM, pH 7.0) containing 0.5 % triton X-100, 2 % (w/v) polyvinylpyrrolidone, 5 mM disodium ethylenediaminetetraacetic acid and 1 mM l-AsA. Homogenates were then centrifuged at 12 000 × g for 20 min at 4 °C and the supernatants used in enzyme assays carried out at 25 °C. The activity of superoxide dismutase (SOD; EC 1.15.1.1) was measured by the method of Beauchamp and Fridovich (1971); catalase (CAT; EC 1.11.1.6) by Aebi (1984); guaiacol peroxidase (GPX, EC 1.11.1.7) by Zaharieva *et al.* (1999); ascorbate peroxidase (APX, EC 1.11.1.11) by Nakano and Asada (1981) and glutathione reductase (GR, EC 1.6.4.2) by Foyer and Halliwell (1976). Enzyme activities were defined as in original methods and expressed as units of enzyme activity per milligram protein, which was estimated according to Bradford (1976).

### Hydrogen peroxide content

Endogenous hydrogen peroxide (H_2_O_2_) was measured using the method of Loreto and Velikova (2001).

### Lipid peroxidation

The extent of lipid peroxidation was estimated by quantifying the malondialdehyde (MDA) content of leaves according to the method of Heath and Packer (1968).

### Statistical analysis

Statistical analysis of the data was carried out using SPSS version 11.0 for windows (SPSS Inc. 2001). Two-way analysis of variances (ANOVAs) were used to determine whether salinity, exogenous treatments and their interactions as grouping factors had significant effect on response variables such as biomass and biochemical parameters. A post hoc Bonferroni test was performed to find significant (*P* < 0.05) differences among individual means of the treatments.

## Results

### Growth parameters

**Figure 1.**
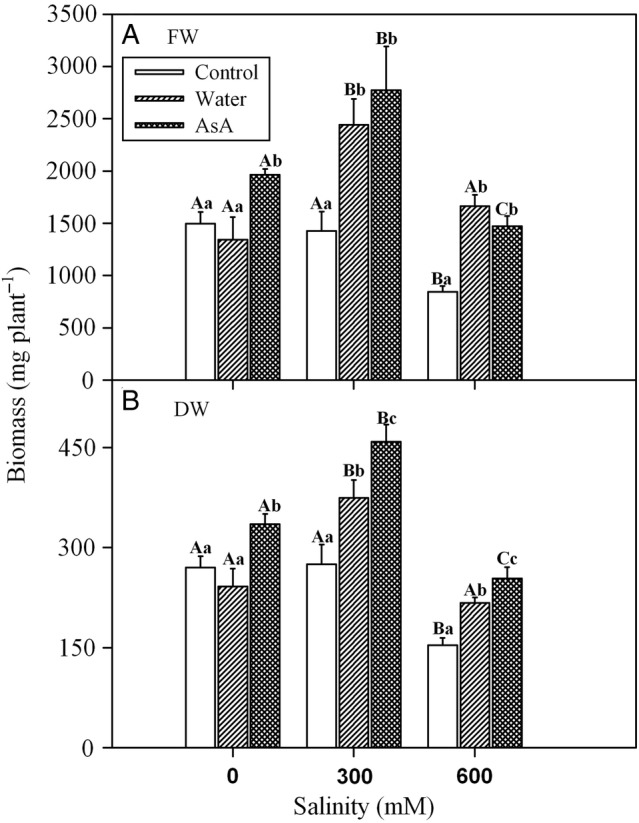
Effect of salinity and exogenous treatments on shoot growth of *L. stocksii* seedlings. Fresh (A) and dry (B) weight values (in mg plant^−1^) are given as bars representing mean ± standard error of four plants. Different capital letters across salinity treatments and small letters within each salinity level are significantly different from each other (*P* < 0.05; Bonferroni test).

Two-way ANOVA indicated significant effects of salinity, exogenous treatments and their interaction on shoot FW and DW of *L. stocksii* (Table 1). Neither FW nor DW of the control (untreated) plants was affected by moderate salinity (300 mM NaCl). However, high salinity (600 mM NaCl) reduced plant growth to ∼50 % in comparison with control (Fig. 1). Exogenous application of water improved FW and DW under saline conditions, while application of exogenous AsA solution ameliorated FW and DW in all salinity treatments. Improvement in plant DW by AsA application was significantly higher than that by water-spray, while FW improvement by AsA and water-sprays were comparable.

**Table 1.** Two-way ANOVA of the effects of exogenous treatments (E), salinity (S) and their interaction (E × S) on different parameters of *Limonium stocksii*. Numbers are the *F*-values with the level of significance given as superscript. *P < 0.05, **P < 0.01, ***P < 0.001 and ns, non-significant.

Parameters	E	S	E × S
FW	13.172***	15.684***	3.312*
DW	23.557***	44.626***	3.432*
Leaf osmolality	3.308^ns^	1.125*	1.480^ns^
MDA	23.069***	28.447***	5.240***
H_2_O_2_	8.254**	1.601*	2.377*
Proline	30.134***	1.593^ns^	3.393^ns^
TSS	970.97***	391.98***	50.981***
AsA	531.19***	688.07***	342.2***
SOD	4.051*	54.321***	2.506^ns^
CAT	1.238^ns^	3.811^ns^	0.920^ns^
GPX	0.529^ns^	23.781***	0.109^ns^
APX	37.139***	29.036***	5.183**
GR	26.738***	19.499***	1.499*

### Leaf water status

**Figure 2.**
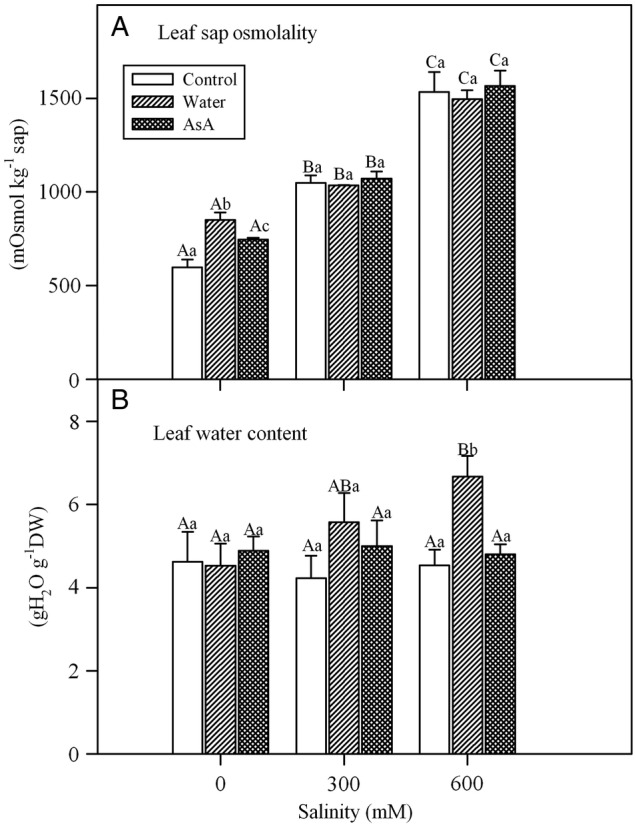
Effect of salinity and exogenous treatments on (A) leaf sap osmolality and (B) water content of *L. stocksii* seedlings. Bars represent mean ± standard error. Different capital letters across salinity treatments and small letters within each salinity level are significantly different from each other (*P* < 0.05; Bonferroni test).

Analysis of variance showed a significant effect of salinity on the osmolality of leaf sap of *L. stocksii* seedlings (Table 1). Increase in salinity caused a rise in osmolality (Fig. 2A), although exogenous treatments did not affect the values of osmolality. Leaf water content was not affected by increases in salinity (Fig. 2B). Exogenous treatments had no effect on leaf water content, except that water-spray at 600 mM NaCl increased water content compared with other treatments and control.

### Osmoprotectants and antioxidants

**Figure 3.**
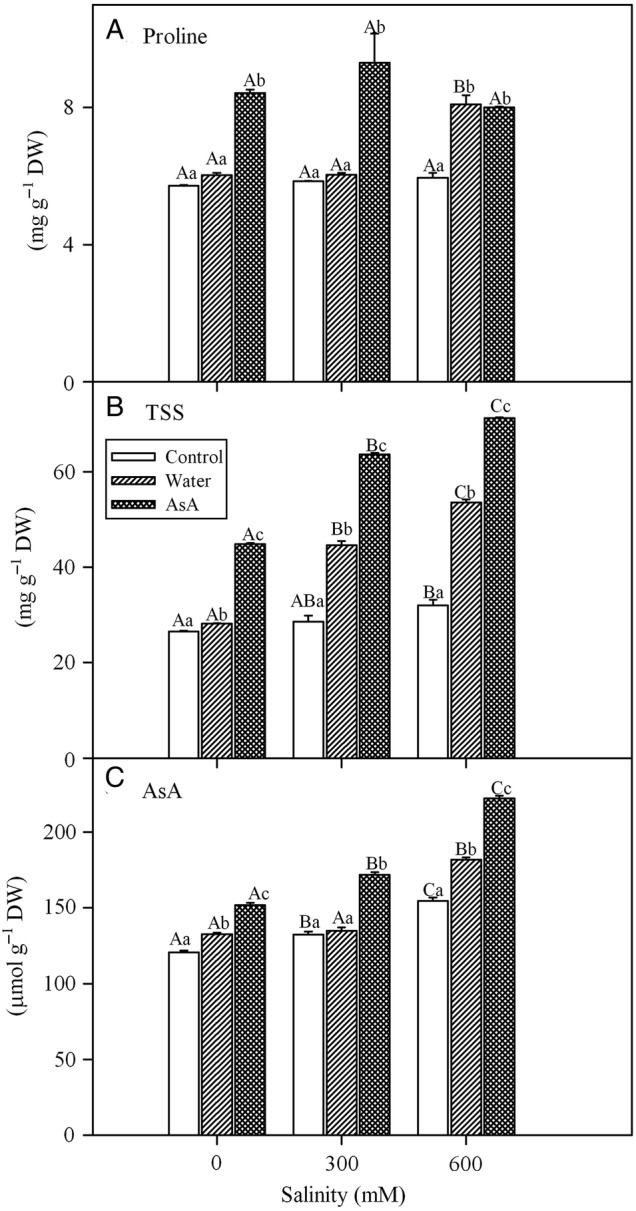
Effect of salinity and exogenous treatments on (A) proline, (B) TSS and (C) reduced ascorbate (AsA) concentrations of *L. stocksii* leaves. Bars represent mean ± standard error. Different capital letters across salinity treatments and small letters within each salinity level are significantly different from each other (*P* < 0.05; Bonferroni test).

Salinity did not affect proline concentrations in *L. stocksii* (Table 1). Exogenous AsA increased proline concentrations of *L. stocksii* seedlings in all salinity treatments while water-spray enhanced proline concentrations only in 600 mM NaCl treatment (Fig. 3A). TSS increased under saline conditions and exogenous treatments resulted in a further increase in the concentration of TSS, with pronounced effects in the case of exogenous AsA (Table 1 and Fig. 3B). Salinity treatments caused an increase in endogenous AsA concentration and exogenous treatments, in particular exogenous AsA, further increased endogenous AsA levels (Table 1 and Fig. 3C).

### Antioxidant enzymes

**Figure 4.**
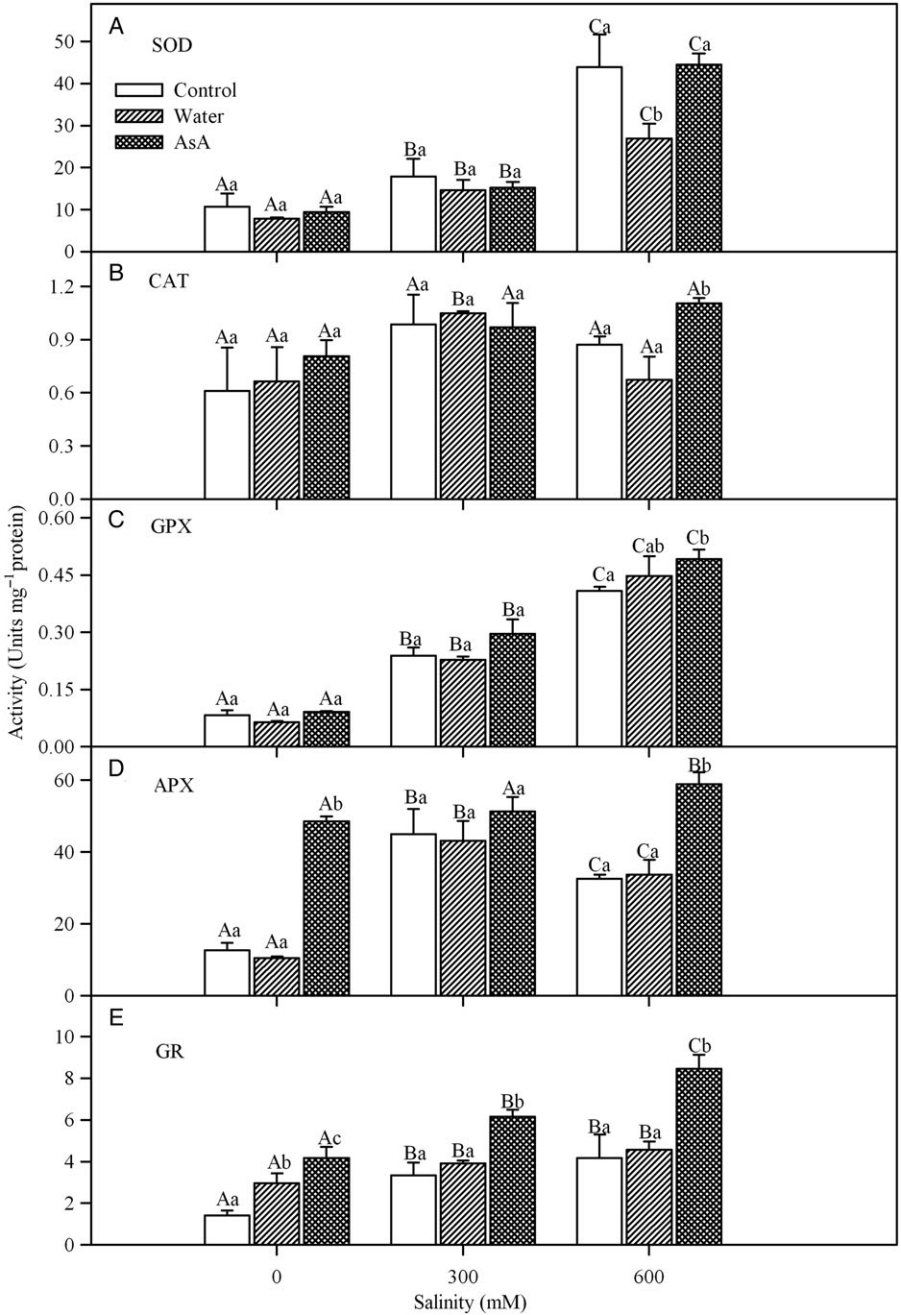
Effects of salinity and exogenous treatments on SOD (A), CAT (B), GPX (C), APX (D) and GR (E) activities of *L. stocksii* leaves. Bars represent mean ± standard error. Different capital letters across salinity treatments and small letters within each salinity level are significantly different from each other (*P* < 0.05; Bonferroni test).

Salinity, exogenous treatments and their interaction had no significant effect on CAT activity (Table 1 and Fig. 4B). However, the activity of SOD increased with increases in salinity (Fig. 4A) although exogenous treatments had no significant effect on SOD activity (Table 1). Likewise, the activity of GPX increased significantly with increases in NaCl concentration (Fig. 4C), but it was unaffected by exogenous treatments and their interaction (Table 1). The activity of APX showed a somewhat different response; activity increased under saline conditions with a maximum value in 300 mM NaCl treatment and exogenous treatment with AsA also caused a significant increase in APX activity (Fig. 4D). Salinity, exogenous treatments and their interaction had a significant effect on GR activity (Table 1) and exogenous AsA caused a substantial increase in GR activity (Fig. 4E).

### Hydrogen peroxide content

**Figure 5.**
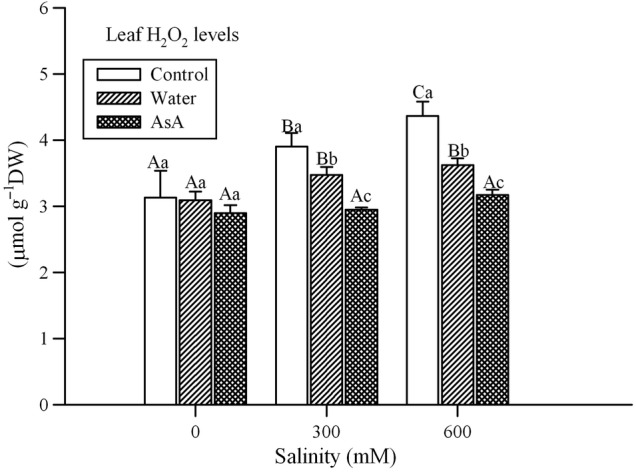
Effect of salinity and exogenous treatments on hydrogen peroxide (H_2_O_2_) concentrations of *L. stocksii* leaves. Bars represent mean ± standard error. Different capital letters across salinity treatments and small letters within each salinity level are significantly different from each other (*P* < 0.05; Bonferroni test).

Analysis of variance indicated a significant effect of salinity, exogenous treatments and their interaction on H_2_O_2_ concentration (Table 1). The concentration of H_2_O_2_ increased with an increase in salinity but exogenous treatments with both water and AsA decreased endogenous H_2_O_2_ levels under saline conditions (Fig. 5).

### Lipid peroxidation

**Figure 6.**
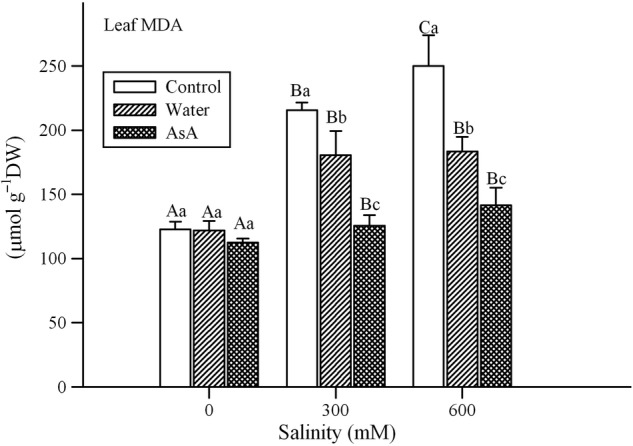
Effect of salinity and exogenous treatments on lipid peroxidation (MDA concentration) in *L. stocksii* leaves. Bars represent mean ± standard error. Different capital letters across salinity treatments and small letters within each salinity level are significantly different from each other (*P* < 0.05; Bonferroni test).

A significant effect of salinity, exogenous treatments and their interaction was found on MDA concentration (indicator of lipid peroxidation) of *L. stocksii* seedlings (Table 1). An increase in salinity resulted in a rise in MDA concentration and exogenous treatments reduced MDA under saline conditions (Fig. 6).

## Discussion

*Limonium stocksii* gained similar biomass over 30 days in 300 mM NaCl as in the absence of salt; however, a further increase in salinity markedly inhibited biomass production. Although growth inhibition occurred at high salinity, *L. stocksii* appears to be more tolerant than other members of the genus: as growth inhibition was observed in *L. perezii* at 110 mM NaCl (Grieve *et al.* 2005) and in *L. sinense* at 300 mM NaCl (Ding *et al.* 2009); growth of *L. pectinatum* was promoted at 100 mM NaCl and was similar to control at 200 mM NaCl (Morales *et al.* 2001). Our results are also similar to growth responses reported for co-occurring halophytes such as *Suaeda fruticosa*, *Arthrocnemum macrostachyum*, *Avicennia marina*, *Aeluropus lagopoides* and *Urochondra setulosa* (Gul and Khan 1998; Aziz and Khan 2000; Khan *et al.* 2000; Gulzar and Khan 2006).

Halophytes utilize internal inorganic ions (Na^+^ and Cl^−^) for osmotic adjustment, by sequestering them in vacuoles with associated synthesis and accumulation of organic/compatible solutes in the cytoplasm (Wyn Jones and Gorham 2002; Flowers and Colmer 2008; Hussin *et al.* 2013). The increase in leaf sap osmolality of *L. stocksii* seedlings with rise in external salinity could be an indicator of such solute accumulation for osmotic adjustment. The linear increase in shoot Na^+^ and Cl^−^ ions in *L. stocksii* seedlings reported in an earlier study (Zia *et al.* 2008) also supports this assumption. Similar results have been reported for co-occurring species such as *S. fruticosa* (Hameed *et al.* 2012), *A. marina* (Aziz and Khan 2000) and *U. setulosa* (Gulzar and Khan 2006). Accumulation of compatible solutes such as choline-*O*-sulfate and betaines of β-alanine, proline and hydroxyl-proline, as reported for taxa within Plumbaginaceae, could also be responsible for osmotic adjustment in our test species (Hanson *et al.* 1994; Gagneul *et al.* 2007; Flowers and Colmer 2008).

Salinity is widely reported to enhance production of ROS (Ozgur *et al.* 2013), which can damage important cell components such as proteins, lipids and nucleic acids (Blokhina *et al.* 2003; Apel and Hirt 2004; Gill and Tuteja 2010). We also observed an increase in H_2_O_2_ concentration (a common ROS) in *L. stocksii* seedlings in response to increasing salinity, as reported for many other halophytes (Jithesh *et al.* 2006; Ozgur *et al.* 2013). Lipid peroxidation, a non-enzymatic autoxidation process due to ROS, is commonly used as a measure of salinity-induced oxidative stress and plant sensitivity (Ozgur *et al.* 2013). It is generally measured in terms of MDA contents, which are common end-products of lipid peroxidation. Generally, a positive linear relationship between MDA concentration and salinity is reported for halophytes (Jithesh *et al.* 2006; Ozgur *et al.* 2013). For instance, MDA concentrations increased with increasing salinity in halophytes such as *Cakile maritima* (Ksouri *et al.* 2007), *Sesuvium portulacastrum* (Lokhande *et al.* 2011), *Gypsophila oblanceolata* (Sekmen *et al.* 2012) and *Sphaerophysa kotschyana* (Yildiztugay *et al.* 2013). We also found a linear increase in MDA concentrations with increasing NaCl treatments in *L. stocksii*. Although the relationship between MDA levels and plant performance is complex (Hameed *et al.* 2012; Alhdad *et al.* 2013), some stress signalling roles of MDA are also emerging (Weber *et al.* 2004). Slightly higher MDA concentrations in *S. fruticosa* (Hameed *et al.* 2012) were associated with better growth.

Halophytes employ a number of enzymatic and non-enzymatic antioxidants to prevent oxidative damage and keep ROS concentrations within a narrow functional rage (Jithesh *et al.* 2006; Ozgur *et al.* 2013). Superoxide dismutase is considered the first line of defence against ROS under stress conditions (Alscher *et al.* 2002). *Limonium stocksii* seedlings under saline conditions showed increased activity of SOD, which was similar to that reported for *Bruguiera parviflora* (Parida *et al.* 2004), *Atriplex portulacoides* (Benzarti *et al.* 2012) and *S. portulacastrum* (Lokhande *et al.* 2011). Catalase activity in *L. stocksii* remained unchanged with the increase in salinity indicating either constitutive expression or importance of other than CAT-based H_2_O_2_ detoxification under salinity stress. Similar CAT activity was also reported for another halophyte *A. portulacoides* in up to 400 mM NaCl salinity (Benzarti *et al.* 2012). Guaiacol peroxidase activity increased significantly in *L. stocksii* seedlings under salinity as in *B. parviflora* under saline conditions (Parida *et al.* 2004). Apart from their role in H_2_O_2_ detoxification, GPXs are also involved in a number of physio-chemical processes such as growth, auxin metabolism, biosynthesis of ethylene and lignin (Lagrimini and Rothstein 1987; Dionisio-Sese and Tobita 1998; Kim *et al.* 1999; Matamoros *et al.* 2003; Jouili *et al.* 2011). Activities of APX and GR were reported to increase in *A. portulacoides* (Benzarti *et al.* 2012), *Salicornia brachiata* (Parida and Jha 2010) and *B. parviflora* (Parida *et al.* 2004) under salinity stress (Jithesh *et al.* 2006). A similar increase in the activities of these enzymes in *L. stocksii* was also observed indicating their role in ROS detoxification under salinity stress.

Many low-molecular-weight antioxidant substances such as AsA are also involved in ROS detoxification in halophytes (Jithesh *et al.* 2006; Hameed and Khan 2011). AsA contents of *L. stocksii* leaves increased under saline conditions similar to *Sphaerophysa kotschyana* (Yildiztugay *et al.* 2013). Increased AsA concetrations in *L. stocksii* could be due to better recycling via the Asada–Halliwell–Foyer pathway and/or its increased biosynthesis. Soluble sugar concentrations of *L. stocksii* leaves increased under saline conditions and this supports a direct relationship between sugar (sucrose) concentration and AsA biosynthesis (Nishikawa *et al.* 2005). Soluble sugars can also act as ROS scavengers (Couée *et al.* 2006; Nishizawa *et al.* 2008; Keunen *et al.* 2013).

Proline accumulation is often used as stress indicator in plants (Kishor and Sreenivasulu 2013; Ben Rejeb *et al.* 2014). However, proline levels remained unchanged in *L. stocksii* seedlings under saline conditions. This may indicate adequate stress management and the absence of injury symptoms also supports this assumption.

Experiments for studying effects of exogenous application of different chemicals such as AsA involve spray of aqueous solutions and any improvement in growth and salt tolerance by such treatments may be due to water rather than the chemical. Hameed *et al.* (2012) showed the importance of such water-spray in salinity tolerance of a coastal halophyte *S. fruticosa*. Water-spray improved sub-cellular defence in *S. fruticosa* probably by mitigating osmotic constraint thereby water acquisition (Hameed *et al.* 2012). Water-spray on *L. stocksii* seedlings resulted in a significant improvement in growth and levels of antioxidant enzymes. This indicates that the improvement in growth by exogenous AsA treatment could partly be due to water in the solution rather than the AsA itself: an increase in leaf water content and levels of TSS and proline and low oxidative damage support this conclusion. However, the DW was greater in the AsA treatment than the water-spray alone at all salinities (Fig. 1), suggesting specific effects of AsA. At high salinity, the TSS concentration and the activities of the antioxidant enzymes were higher with the AsA-treatment than with the water-spray alone (only for GPX the difference was not significant). The proline concentration was higher in the AsA treatment than with the water-spray at the 300 mM treatment, but not at the higher salinity. Based on our results, it appears that AsA does bring about specific improvements to growth and these were associated with biochemical changes in the leaves. Similar results for the treatment with AsA have been reported for *S. fruticosa* (Hameed *et al.* 2012) and in many non-halophytes (Shalata and Neumann 2001; Dolatabadian *et al.* 2008; Younis *et al.* 2010). Exogenously applied AsA is easily absorbed, transported and metabolized in plants (Sapers *et al.* 1991; Franceschi and Tarlyn 2002), where it has a variety of metabolic and physiological functions in antioxidant defence, photosynthesis, transmembrane electron transport, biosynthesis of plant hormones and/or cell expansion (Conklin *et al.* 1996; Arrigoni and De Tullio 2000, 2002; Khan *et al.* 2011).

Exogenous application of AsA decreased lipid peroxidation in *L. stocksii* seedlings under salinity as reported for *S. fruticosa* (Hameed *et al.* 2012) and in crops such as *Brassica napus* (Dolatabadian *et al.* 2008) and *Phaseolus vulgaris* (Saeidi-Sar *et al.* 2013), indicating better antioxidant defence. Shalata and Neumann (2001) pointed out that the protective effects of exogenous AsA appeared to be related to its antioxidant activity rather than its possible use as an organic substrate for respiratory metabolism. Exogenous AsA significantly increased levels of endogenous AsA and Asada–Halliwell–Foyer pathway enzymes in *L. stocksii* seedlings compared with both un-sprayed and water-sprayed plants. Likewise, exogenous AsA also increased APX and GR activities in *Vicia faba* seedlings compared with control plants (Younis *et al.* 2010). These findings show that the exogenous AsA improves salinity tolerance through stimulating antioxidant defence.

## Conclusions

**Figure 7.**
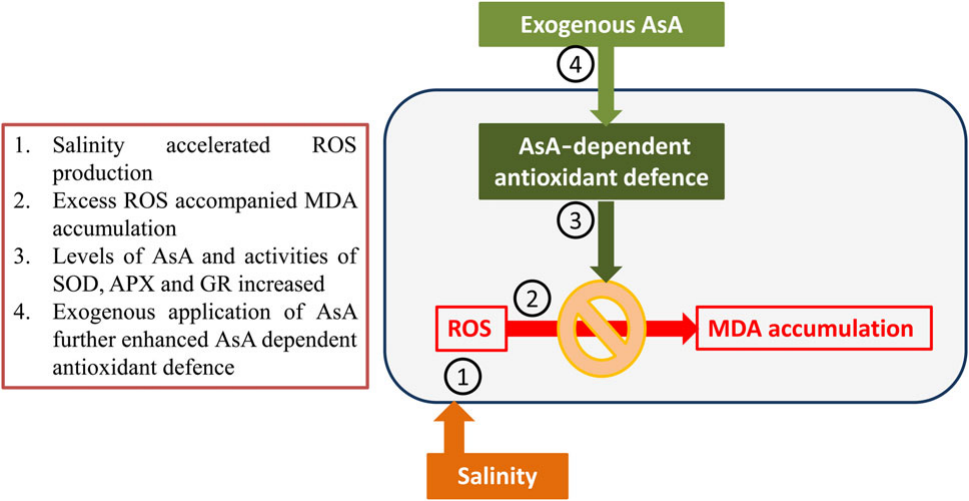
Pictorial description of key findings.

Shoot growth of *L. stocksii* plants was inhibited at high salinity and coincided with higher levels of MDA. Leaf osmolality progressively increased to maintain osmo-balance. AsA-dependent antioxidant enzymes contributed to the salinity resistance as evident by increasing level of AsA and activities of Asada–Halliwell–Foyer pathway enzymes (Fig. 7). Exogenous AsA improved the functioning of AsA-dependent antioxidant system.

## Sources of Funding

The authors thank Higher Education Commission of Pakistan for provision of funds under a research grant entitled ‘Salt-induced Oxidative Stress: Consequences and Possible Management’.

## Contributions by the Authors

Obtaining funds: M.A.K.; experiment designing: A.H., B.G., M.A.K.; execution of experiments: A.H., T.H.; data analyses: A.H., S.G., I.A., B.G.; paper writing: M.A.K., A.H., S.G., I.A.

## Conflicts of Interest Statement

None declared.
